# Numerical simulation of wind field and sand flux in crescentic sand dunes

**DOI:** 10.1038/s41598-021-84509-x

**Published:** 2021-03-02

**Authors:** Huiwen Zhang, Zhen Wu, Jing Hu, Zhiping Zhang, Bin Xiao, Jianping Ma

**Affiliations:** 1 State Key Laboratory Breeding Base of Desertification and Aeolian Sand Disaster Combating, Gansu Desert Control Research Institute, Lanzhou, 730070 China; 2 Lanzhou Geophysical National Field Scientific Observation and Research Station, Earthquake Administration, Earthquake Administration of Gansu Province, Lanzhou, 730000 China; 3 Key Laboratory of Loess Earthquake Engineering, China Earthquake Administration, Lanzhou, 730000 China

**Keywords:** Environmental sciences, Natural hazards

## Abstract

Sand flux is the key factor to determine the migration of sand dunes and the erosion to the surrounding environment. There are crescent-shaped sand dunes of various scales in the desert, and there are significant differences in spatial wind field and sand flux among them. However, due to the difficulty of monitoring, it is difficult to continuously observe the spatial wind field and sand flux around the larger crescentic dunes. On the basis of the Reynolds-Average Navier–Stokes (RA-NS) equation and the stress and sand flux model, the distribution of wind field and sand flux of a circular dune with a height of 4.2 m and a length of about 100 m during the four evolutionary periods of the evolution into a crescentic dune was simulated in this study. By comparing with the measured results, we verified that the closer to the leeward side, the more the simulated values of the velocity in wind field and sand flux were in line with the measured results. In order to further analyze the influence of the height of dune and other relevant parameters on sand flux, we simulated the influence on wind field and sand flux by changing the air viscosity and wind velocity of upper boundary. We found that the air viscosity mainly affected the amount of deposited sand on the leeward side of sand dune, while the increase of wind velocity would undoubtedly increase the sand flux of the whole sand dune. In addition, the simulation results also showed that the influence of changes in height of dune on the turbulent intensity of leeward side was very significant, and the turbulent intensity increased with the height of dune. The height changes of tall dunes gradually affected the transport of sand caused by wind flow behind the leeward side because that the rotation of the wind flow would form new vortexes at the large pores behind the leeward side, which would increase the turbulent energy in space and thus would increase the distance of migration of the lifting sand. While the low sand dunes could not form extra small vortexes at the bottom of the leeward side, so the wind velocity was small and the eddy currents behind the leeward side were more stable. The simulation results indicated that wind velocity was not the only reason for increasing the amount of sand flux, and the fluctuation of wind flow caused by turbulence could also stimulate the movement of sand particles on the ground.

## Introduction

Understanding the migration mechanism and the deposition process of crescentic dunes have important guiding role in preventing and controlling wind-sand disasters^1–4^. Under the action of unidirectional wind, sand dunes of different scales have great differences in sand flux and spatial distribution of wind field^5–7^. Moreover, the differences in recharge of sand source and wind condition can also lead to the differences of spatial sand flux^8,9^, so many of the larger crescentic dunes near the oasis edge pose a potential threat to the sounding environment.

It is well known that the structure of wind-sand flow is strongly influenced by the properties of underlying surface. Some studies have found that the sand content in the wind-sand flow decreases exponentially with the increase of height^10–12^. However, some other studies have shown that the sand flux presents a square root decreasing relationship with height^13,14^. The results of the previous studies have been obtained through measured observations, and have not yielded uniform conclusions^15,16^. Therefore, the present observation conclusion is that there is a large spatial heterogeneity of sand flux and wind field distribution in different locations and heights of crescentic dunes. In order to obtain the specific understanding of the sand flux and wind field distribution in different locations of crescentic dunes, it is necessary to carry out continuous observation of the wind field around the dunes of different scale.

Unfortunately, larger-scaled observations of crescentic dunes greatly increase the work load of field monitoring. Moreover, conventional observations are not only burdensome but also lack continuity in spatial observations. Althoughmany scholars have used more convenient methods and means to carry out researches on migration and deposition of sand dunes, such as wind tunnel experiments^17–20^, remote sensing survey^21–23^ and so on, it is still difficult to continuously show the variation of wind field and sand flux at different positions and heights of sand dunes of different scale, and it is also difficult to give the detailed structure of wind flow field and the distribution of shear stress in continuous space. To compensate for this defect, many scholars began to use numerical simulation to analyze the changes of wind flow field in sand dunes^13,24–28^. Most of the previous numerical methods are used to simulate smaller dunes, because the simulation of small dunes take a short time, which mainly to solve the problem that the turbulence behind the leeward side is not separated, and then to obtain the disturbance characteristics of shear stress and wind field. However, due to the changeable turbulence of the leeward side in the actual situation, this numerical method can not accurately reflect the influence of turbulence.

With the improvement of computing efficiency of computers in recent years, many scholars have begun to use complex models such as Reynolds-Average Navier–Stokes (RA-NS) equation to simulate the changes of wind flow field^29–33^. Many equations are applied to viscous fluid^1,29,34,35^ and many simulation equations about pure wind field are applied to sand migration and erosion^36–38^. But until now, it has not been reported to carry out a combinatorial continuous simulation study on wind velocity, wind field, spatial sand flux and their influencing factors of crescentic dunes. The aim of our study was to fill this gap. Firstly, the purpose of this study was to evaluate the simulation effect of the RA-NS equation on two-dimensional wind field and flow field, and secondly, to analyze the spatial relations and influencing factors of wind flow field and sand flux. To achieve these goals, three problems were analyzed by numerical simulation in this study: (1) Changes of wind field and sand flux around the sand dune during evolution; (2) Influence of different heights of sand dune and turbulences behind the leeward side on sand flux; (3) Variation in the amount of sand flux caused by the height of dune and wind velocity of boundary. The results of this study could provide a theoretical reference for the researches on surface erosion and migration of crescentic sand dunes of different scale.

## Research methods and numerical simulations

### Simulation of wind flow

Because of the complexity of turbulence in the wind field, we used the RA-NS equation^29,39^ to simulate the wind field, which performs well in the fluid simulation. RA-NS equation is widely used in various engineering problems, including compressed flow, two-phase flow, uncompressed flow of constant matter and so on^40^. The related simulation theory is called the theory of turbulent mode. The theory assumes that the variables in turbulent flow field are composed of a time-averaged variable and a pulsating quantity variable, and then a NS equation with uniform variation on the time scale can be obtained. The specific methods are as follows: the various physical parameters of fluid are expressed as stable pulsating values, and some parameters can also be averaged, then the governing equation of the statistical average physical quantities can be obtained. The continuous equation and the momentum equation are shown as follows:

1 $$\frac{{\partial {\rho }}}{{\partial {\text{t}}}} + \frac{{\partial \left( {{\rho u}_{{\text{i}}} } \right)}}{{\partial {\text{x}}_{{\text{i}}} }} = 0$$

2 $$\nu_{x}^{^{\prime}} \frac{{\partial \nu_{x}^{^{\prime}} }}{{\partial x^{\prime}}} + \nu_{y}^{^{\prime}} \frac{{\partial \nu_{x}^{^{\prime}} }}{{\partial y^{\prime}}} = - \frac{{\partial p^{\prime}}}{{\partial x^{\prime}}} + \frac{1}{{\text{Re}}}\left( {\frac{{\partial^{2} \nu_{x}^{^{\prime}} }}{{\partial {\text{x}}^{^{\prime}2} }} + \frac{{\partial^{2} \nu_{x}^{^{\prime}} }}{{\partial {\text{y}}^{^{\prime}2} }}} \right)$$

3 $$\nu_{x}^{^{\prime}} \frac{{\partial \nu_{x}^{^{\prime}} }}{{\partial x^{\prime}}} + \nu_{y}^{^{\prime}} \frac{{\partial \nu_{x}^{^{\prime}} }}{{\partial y^{\prime}}} = - \frac{{\partial p^{\prime}}}{{\partial x^{\prime}}} + \frac{1}{{\text{Re}}}\left( {\frac{{\partial^{2} \nu_{y}^{^{\prime}} }}{{\partial x^{^{\prime}2} }} + \frac{{\partial^{2} \nu_{y}^{^{\prime}} }}{{\partial y^{^{\prime}2} }}} \right)$$

where $R{\text{e}} = \frac{{\rho {\text{D}}}}{\nu }{\text{V}}_{{\text{r }}}$, *D* is the diameter of the sand particles, $v$ = 1.46 × 10^–5^ m^2^ s^−1^. $\nu { }$ represents the viscous coefficient of the air. *u* represents the calculated mean velocity of wind. $V_{r} { }$ is the relative velocity between sand particle and wind field. To obtain accurate and stable results, constant uniform wind field was used in this study.

### Simulation of sand flux

Wind in different directions will cause different shear stress on the surface of sand dunes, and the amount of sand flux is mainly affected by the magnitude of the shear stress. The greater the shear stress, the greater the amount of sand flux. Burkow equation was used to describe sand flux in this study^41,42^:

4 $$\frac{{\partial {\text{h}}}}{{\partial {\text{x}}}} + \nabla_{{\left( {xi,yi} \right)}} \cdot q\left( {\tau \left( u \right)} \right) = 0$$

where *q* is a relation function between transmission rate and shear stress. Because shear stress is related to the velocity of wind, so *q* can be expressed as follows:

5 $${\text{q}}_{s} = \varepsilon \sqrt {\left( {s - 1} \right)gd_{s}^{3} } \cdot \left( {\frac{4\tau \left( u \right)}{{\rho_{f} \left( {s - 1} \right)gd_{s} }} - {\tau }_{c} } \right)^{\frac{3}{2}}$$

6 $$\tau \left( {\text{u}} \right) = \frac{1}{8}\rho_{f} f\left| u \right|^{2}$$

where ${ }\rho$ denotes the sediment density, $d_{s}$ is the median scale of sand particles (3 mm), $\tau_{c} { }$ is the critical shear stress (the value of it is 0.05). $s = \rho_{s} /{ }\rho_{f} { }$ with $\rho_{f}$ being the fluid density. The value of $\varepsilon$ is 10^–8^. The $f$ in Eq. (6) is expressed as 64/Re.

### The interaction force between particles and fluid

In general, moving sand particles are subjected to drag force of airflow, electrostatic force and gravity in the wind field. Among them, the drag force of air flow and the gravity of sand particles have the greatest influence on the motion of sand particles. This study ignored the influence of electrostatic force. And the other parameters are expressed as follows.

For nearly spherical sand particles, their gravity $F_{g}$ can be expressed as follows:

7 $${\text{F}}_{g} = { }\frac{1}{6}\pi \rho_{g} {\text{D}}^{3} g$$

where ${ }\rho_{g}$ is the density of sand particles, *D* is the diameter of sand particles, *g* is the acceleration constant of gravity.

According to the research of Anderson and Haff^43^, the drag force of wind field on sand particles can be expressed as follows:

8 $${\text{F}}_{D} = \frac{1}{8}C_{D} \pi {\text{D}}^{2} \left| {V_{r} } \right|V_{r}$$

where $V_{r}$ is the relative velocity between sand particles and wind field, which can be expressed as follows:

9 $${\text{V}}_{r} = \sqrt {\left( {u - u_{D} } \right) + \left( {v - v_{D} } \right)}$$

where ${ }u_{D}$ and $v_{D}$ represent the velocity of sand particles in the horizontal and vertical directions, respectively. $C_{D}$ is the resistance coefficient, which can be calculated by the following empirical equation:

10 $${\text{C}}_{{\text{D}}} = (0.63 + \frac{4.8}{{{\text{Re}}^{\frac{1}{2}} }})^{2}$$

where *R*e denotes the Reynolds coefficient. The fluid pressure gradient causes shear stress in the velocity direction, resulting in an updraft. It can be expressed as follows:

11 $${\text{F}}_{l} = \frac{1}{8}\pi \rho_{a} C_{l} {\text{D}}^{2} \left( {u_{up}^{2} - u_{down}^{2} } \right)$$

where ${ }u_{up}$ and $u_{down}$ are the wind velocity of sand particles at the upper and lower boundary, respectively. And $C_{l }$ is the rising force coefficient, which value is 0.85 times of $C_{D}$. So the migration equation of sand particles can be written as follows:

12 $${\text{m}}_{{\text{p}}} \frac{{{\text{dU}}_{{\text{D}}} }}{{{\text{dx}}}} = {\text{F}}_{{\text{g}}} + {\text{F}}_{{\text{D}}} + {\text{F}}_{l}$$

where ${ }m_{p}$ is the mass of sand particle.

### Simulation of sand flow deposition and morphological evolution

According to Eq. (4), the shear stress of wind on the surface of sand dunes has a threshold value^44^, that is, the maximum amount of sand flux that wind can carry. Burkow equation also can be writen to describe the morphological changes of sand dunes.

13 $${\rho }_{{\text{s}}} \frac{{\partial {\text{h}}}}{\partial x} + \nabla q = 0$$

where ${ }\rho_{s} { }$ is the density of sand flow phase, *h* = *h* (x, y, t) and *q* = *q* (x, z, t) are height of dune and total vertically intergrated sand flux. The sand transportation can be described in terms of two species, saltation and reptation. An empirical equation^45^ was used to describe the effect of wind velocity on the leaping sand flow here:

14 $$ {\text{q}}_{sat} = \left\{ {\begin{array}{ll} {C_{sat} \frac{{\rho_{g} }}{g}u_{*} \left( {u_{*} + 7.6u_{t} + 2.05} \right) } & \quad {u_{*} > u_{t} } \\ 0 & \quad {u_{*} \le u_{t} } \\ \end{array} } \right. $$

where $C_{sat}$ is the correction factor with a value of 0.5. *g* is the acceleration constant of gravity. $u_{*}$ and *u*_t_ are the surface sliding velocity and critical sliding velocity, respectively. When sand particles move on the surface of sand dunes, collision between sand particles often occurs, and the slope will greatly affect the trajectory of sand particles. Considering the relationship between sand particles and slope, the sliding velocity threshold can be expressed as follows:

15 $${\text{u}}_{t} = \sqrt {\left( {\rho_{m} - \rho_{g} } \right)gd_{s} \left( {cos\theta + \frac{sin\theta }{{tan\theta_{rep} }}} \right)/\rho_{g} } /10$$

where ${ }d_{s}$ is the average size of sand particles, $\theta$ is the angle of slope, $tan{\theta } = \nabla {\text{h}}$ and $\theta_{rep}$ is the angle of repose, $\theta \approx 37^\circ$ and this value refers to Iversen’s finding^46^.

The creep of sand particles is caused by the collision of sand particles moving on the surface, so the reptation and the jump of sand particles are proportional. According to previous studies, the reptation of sand particles can be expressed as follows^47^:

16 $${\text{q}}_{rep} = (1 + \alpha )q_{sal} e_{x} - \alpha \beta q_{sal} \nabla h$$

where the coefficients ${ }\alpha$ and $\beta$ represent the fraction of the total sand flux.

Considering the transition of sand flow from unsaturated to saturated state, the change of sand flux can be expressed as follows:

17 $$\nabla {\text{q}}_{sal} = \left( {q_{sat} - q_{sal} } \right)/l_{sat}$$

where $q_{sal}$ is the saltation flux, and $l_{sat}$ is the saturation length, which have a relationship with friction velocity^48^.

18 $$l_{sat} = al_{drag}$$

where the parameter *l* represents the distance through which the sand grain reaches the wind velocity from standing at a site.

Except for considering the migration of dune, the avalanche phenomenon on the surface during the evolution can be explained by the slope angle. This avalanche phenomenon is closely related to the height of sand dune. We used a diffusion equation combine with a mass balance equation to calculate mass accumulation together. This diffusion equation can be written as follows:

19 $${\text{q}}_{{{\text{ava}}}} = - {\rho }_{{\text{s}}} {\epsilon }\nabla {\text{h}}$$

where $\varepsilon$ is a adjustment factor, and its value is related to the change rate of the height of the dune and the grid size of the model. It is also necessary to define the collapse angle $\theta_{{\text{a}}} { }$. According to the previous research methods^49^, we set that when the slope of the dune surface exceeded 34º, that is, when $\theta_{{\text{a}}} < \theta_{{{\text{rep}}}}$, there would be no avalanche or slippage movement. In this case $q_{{{\text{ava}}}}$ = 0, otherwise, the sand would be redistributed according to the sand flow:

20 $$ \varepsilon  = \left\{ {\begin{array}{ll}    {\frac{{E\left[ {\tanh \left( {\left| {\nabla h} \right|} \right) - \left( {tan\theta _{{rep}} } \right)} \right]}}{{\left| {\nabla h} \right|\rho _{s} }}} &\quad {\left| {\nabla h} \right| > tan\theta _{{rep}} }  \\    0 &\quad {\left| {\nabla h} \right| \le tan\theta _{{rep}} }  \\   \end{array} } \right. $$

For a sufficiently large coefficient *E*, the slope is relaxed independently of *E*. The value of *E* is set as − 0.9. This condition first runs under a constant condition and finally reaches a stable state. Because of the different period of reconstruction caused by the slope of the leeward side, the setting of the critical slope of the leeward side will greatly affect the shape of the longitudinal section of the whole dune.

Combined the previous Eqs. (13)–(19) with the collapse conditions, we could obtain the evolutionary process of surface height of dune until this maximum collapse angle was reached.

Combined with Eq. (10), the total mass equilibrium equation is obtained as follows:

21 $$\frac{{\partial {\text{h}}}}{{\partial {\text{x}}}} + \nabla \cdot uh = \nabla \cdot {\epsilon}\nabla {\text{h}}$$

### Boundary conditions

In the actual situation, the velocity of wind with height may appear as pulsating wind field, but the pulsating wind field will bring great difficulty and uncertainty to the optimization of parameters and comparison with the measured data (the simulation results of pulsating wind field are different each time). Therefore, the upper boundary condition was set to a constant wind velocity, and the left and right boundary conditions were all set to the wind velocity at different heights under the initial conditions. Since the wind field was far from the dune in space, the initial vertical velocity was set as 0. Moreover, it was assumed that the temperature would not cause the air flow to rise.

22 $$\frac{{\partial {\text{u}}}}{{\partial {\text{x}}}} = 0$$

We used an empirical equation to express the friction velocity on the surface of sand dune^50^. The changes of sand particles caused by friction and collision were roughly described by shear stress and density distribution of sand particles:

23 $${\text{u}}_{{{\text{Friction}}}} = \sqrt {{\tau}/{\rho }}$$

In the source direction of the wind, we set the velocity distribution of the wind with the height as follows:

24 $${\text{u}}\left( {\text{z}} \right) = \frac{{{\text{u}}_{{\text{f}}} }}{{\text{k}}}{\text{ln}}\left( {\frac{{\text{z}}}{{{\text{z}}_{0} }}} \right)$$

where $u_{{\text{f }}}$ is the sliding speed, *k* is the Karman constant, and its value is set to 0.41. $z_{0}$ represents the surface roughness of sand dune, and its value is set to d/30. *d* is the diameter of sand particles.

The bottom interface is the contact interface between the sand dune and the ground, where we set it to be no sliding.

The initial morphology of the sand dune was set to a circular dune with a height of 4.2 m, a length of about 100 m and a bottom diameter of 6 m. The diameter of sand particles was set to 200 µm, the density of sand particles was set to 1.2 kg m^−3^, the viscosity of wind was set to 1.46 × 10^–5^ m^2^ s^−1^, the iteration time of wind field and the migration of dune was set to 1 × 10^–4^ s. The whole grid was set to 180 × 50 × 100, and the convergence condition was set to converge when the following conditions were reached.

25 $$\frac{\|{u_{i}^{^{\prime}} - u_{i - 1}^{^{\prime}}\| }}{\|{u^{\prime}}\|} < 10^{ - 5}$$

### Numerical iterative calculation

Equation (20) is equivalent to the momentum equation of a gas state, and it also includes the continuity equation of the variation of the height of the dune. The instantaneous diffusion of sand particles and continuous height change of sand dunes can be obtained by this equation. The discrete form of the equation adopts the steady state spatial difference form, the difference form adopts the central difference form, and the iteration of the whole equation adopts the fully implicit scheme, which overcomes the possible non-convergence in the calculation. Complete a change process of a sand dune can be summarized as four steps: (1) to calculate the spatial wind field, set the time interval as ∆*t* to obtain the spatial wind field distribution; (2) to calculate the sand flux caused by wind velocity and the spatial distribution of sand particles; (3) to calculate the shape and height changes of different position of sand dune after calculating three time intervals because that it takes a certain time from take-off to deposition of sand particles; (4) to obtain the changes of wind field in the new dune shape by substituting the new height and the coordinates of the dune shape into the wind field iterative calculation. And then an iterative process is completed. The scale of time and changes of dune in the simulation calculation are actual time and length.

## Results and discussion

### Simulated variation of wind field in the central axis of crescentic dune

**Figure 1:**
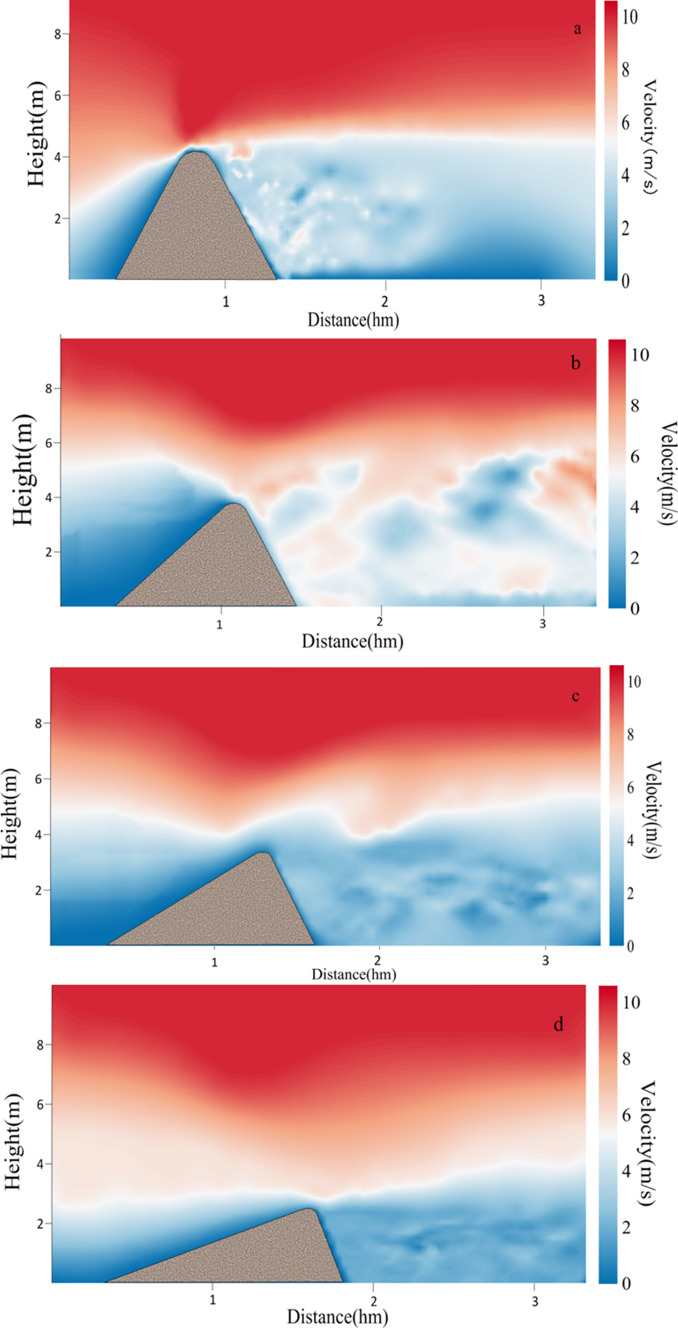
The simulated two-dimensional wind fields at the position of the central axis during the evolution into a crescentic dune. (**a**–**d**) represented the two-dimensional wind field at the position of the central axis of the four stages, respectively. The map was plotted using Matlab 2014a (https://www.mathworks.com).

To better show the distribution changes of wind field and flow field of the leeward side of sand dunes, the longitudinal resolution of the two-dimensional wind field map was added in the models of this study, that is, the horizontal and vertical coordinates showed different spatial scales. Figure 1a showed the simulated wind field at the initial stage of the circular dune. The wind velocity at the windward crest was close to the velocity of the wind flow at the corresponding height due to the blocking effect of the windward side on the wind flow^31^. Velocity of wind field increased with the height from the windward toe to the windward crest. Subsequently, disturbance of the wind field caused by turbulence occurred on the leeward side, while a clockwise vortex was usually formed behind the leeward side as shown in Fig. 3a. However, the eddy current behind the leeward side caused the sand particles on the surface to be blown back to the leeward side, and the upper part of leeward side gradually became steeper, which made the velocity distribution of wind field after the leeward side different greatly and formed a new spatial distribution of two-dimensional wind field (Fig. 1b). From the velocity field, it could be seen that under the combined action of uneven spatial wind velocity and air viscous force^5^, a large number of irregular turbulence appeared behind the leeward side of sand dune, which was close to the result of Zhang^51^. Then the occurrence of these large irregular turbulences would make the sand lifting and deposition of the ground after the leeward side more complicated. Under the continuous action of wind flow, the sand on the windward side was constantly taken away and the windward side slowed down slightly (Fig. 1b). The turbulent flow behind the leeward side continuously carried the sand particles of the leeward toe blowing back to the upper part of the leeward side, which would cause the sand particles of the upper part to accumulate to a certain extent and then collapse down. Obviously, the material reduction at the leeward toe increased the drop rate of the sand particles on the upper part. Through the continuous collapse cycle, the height of the dune gradually decreased, and the slope of the leeward side gradually became gentle with the collapse and accumulation of the sand particles. At this stage (Fig. 1c), the wind velocity at the top of the leeward side decreased as the height of the dune decreased, and the field of wind flow behind the leeward side slowly showed a relatively steady change, then the difference of spatial wind velocity decreased gradually. The erosion of leeward side would continue until the slope reached a certain degree of gentle, that is, when the highest point of sand dune was gradually reduced to the critical height of the leeward side under wind erosion (Fig. 1d), the shear stress of turbulence to the leeward side began to weaken, and then the fluctuation of wind velocity behind the leeward side was further weakened. Now the wind velocity of leeward side was smaller than that of windward side at the same height. On the one hand, as the height of the dune decreased, the wind with lower velocity in the low altitude could easily pass through the top of the dune. On the other hand, after the wind velocity at the top of the leeward side decreased, the resulting turbulent energy also decreased, and the disturbance to the air flow decreased. So the difference of wind velocity behind the leeward side decreased and the overall turbulent phenomenon weakened. The above results showed that the height of sand dune had a significant effect on the turbulent intensity of leeward side. Anyway, although there showed a complicated wind velocity field behind the leeward side, the structure of eddy current behind the leeward side could be seen from the distribution of wind velocity field.

**Figure 2:**
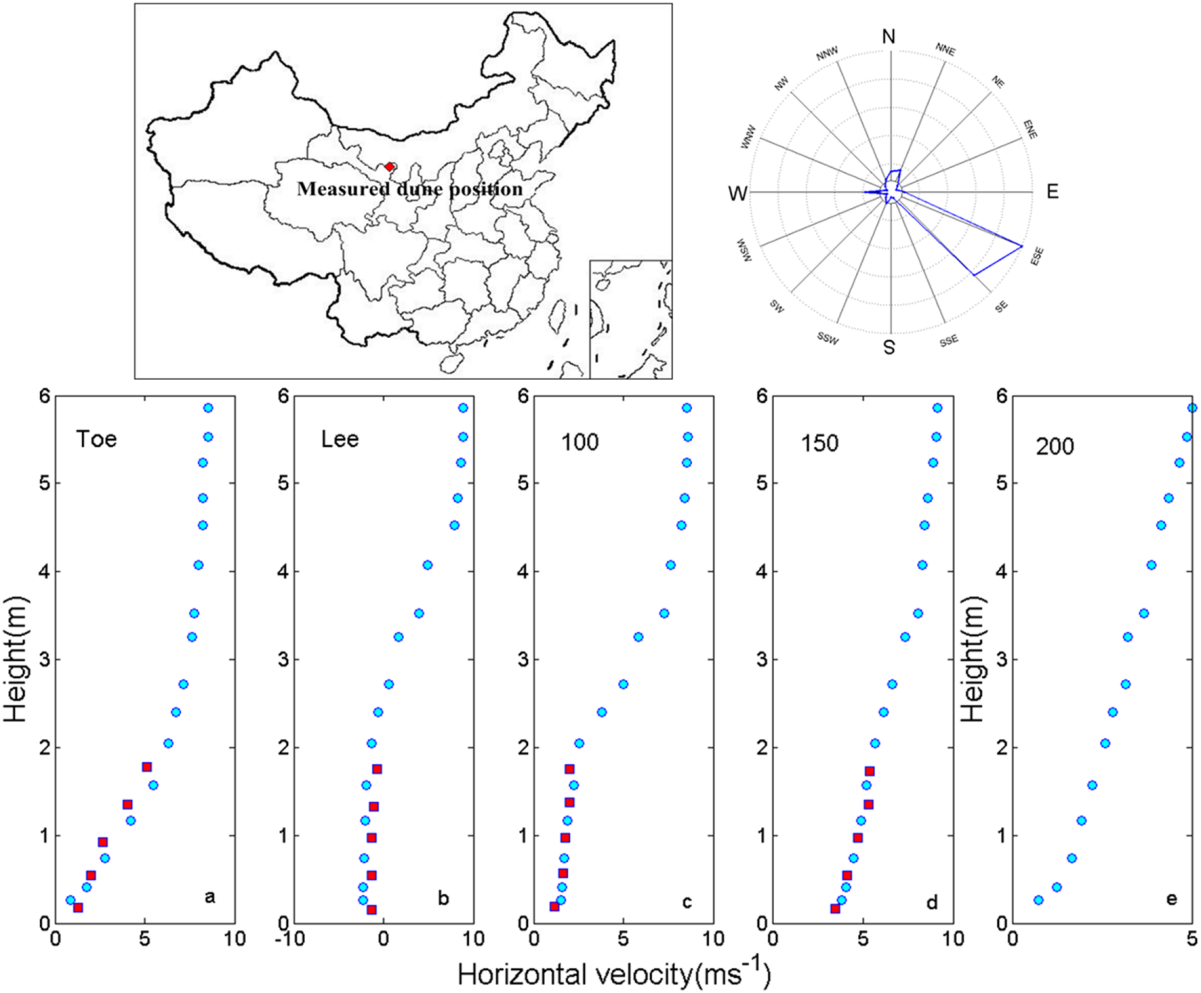
Comparison of simulation in wind field (round blue dots) and field measured wind velocity (square red blocks) at different locations of sand dune (the wind velocity position corresponded to the different distance position in Fig. 1b). The upper right corner showed the wind frequency map of the measured sand dune position. The map was plotted using Matlab 2014a (https://www.mathworks.com) and Surfer 16 (https://www.goldensoftware.com/).

To analyze the vertical distribution of velocity field at different positions of the dune and verify the reliability of the simulation results, we compared the simulated the variation of horizontal wind velocity with height at the windward toe, the leeward toe and the different distance positions at the downwind (Fig. 2). The measured data were from a crescentic dune in the desert of northwest Minqin County of China, which had a length of 86.5 m, a height of 3.7 m, and a maximum wind velocity of 11 m s^−1^. Although the local wind velocity and wind direction had some fluctuations, the wind velocity in the main wind direction was relatively stable.

The simulation results of five positions in Fig. 2 were in good agreement with the measured data, which indicated that the model could reproduce the actual distribution of wind velocity more truthfully. There was significant negative velocity at the leeward side in Fig. 2b which suggested that the eddy current caused by the decrease of pressure at the leeward side would cause the inversion of the horizontal wind field, and which accorded with the conclusions of most of the previous studies^52–54^. In the downwind direction, with the increase of the distance from the leeward side, the influence of eddy current on wind velocity and wind direction decreased gradually, so the vertical wind velocity in the downwind direction tended to be close to the parabola shape again.

**Figure 3:**
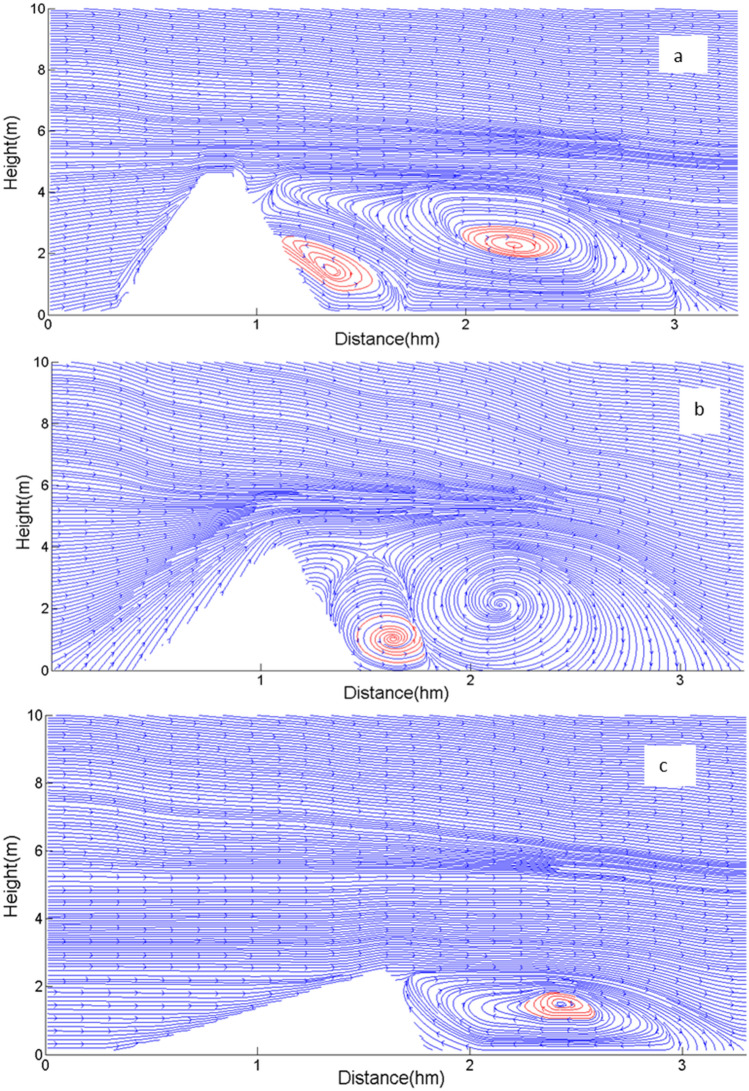
Distributions of two-dimensional wind flow field around the crescentic dune in Fig. 1a (**a**), 1b (**b**) and 1d (**c**) (red circles represented the vortexes).

To further verify the reliability of the simulation, we obtained the distribution maps of two-dimensional wind flow field of sand dune corresponding to Fig. 1a,b,d by calculating the divergence of velocity. From the change of wind flow field in Fig. 3a, it could be seen that the blocking of the air flow by the tall dune made the low pressure zone formed behind the leeward side. Firstly, the wind blowing through the top squeezed the air behind the leeward side to sink. Then the downdraft contacted the ground to form shear force, prompting the air flow to reverse. The recirculation flow was then lifted along the leeward side to fill the low pressure zone formed by the lost air flow. These compressed and refluxed air flows formed a large vortex, with a wide range of spread, whose height was basically the same as that of the resulting dune, and whose length was even larger than that of the resulting dune. This large vortex maintained a near-circular shape, driving the surrounding air to move and then rise along the leeward side. After that, the edge of vortex was in contact with the upper part of the leeward side, so a certain space after air loss was left between the edge of the vortex and the leeward toe, which led to the generation of the second small vortex.

Figure 3b was the wind flow field pattern corresponding to the dune morphology in Fig. 1b. From it we realized that compared with the first stage, the vortex center after the leeward side continuously produced the outflow of the central airflow, while in the first stage the airflow only rotated with the vortex current. This situation would lead to the formation of a low pressure zone in the internal vortex center, and which was easier to accelerate the flow of wind. Moreover, it would also promote the transport of sand particles. Figure 3c displayed the distribution of two-dimensional flow field of dune in Fig. 1d. We found that there was only one large vortex behind the leeward side. The large vortex, though long in length, had been compressed into an ellipse. There was little space between the edge of the vortex and the leeward toe to produce additional vortexes, so there was only one larger vortex in Fig. 3c. Therefore, the different height of dune would produce different number and scale of vortexes, thus affecting the scale of sand flux.

### Simulation of sand flux

**Figure 4:**
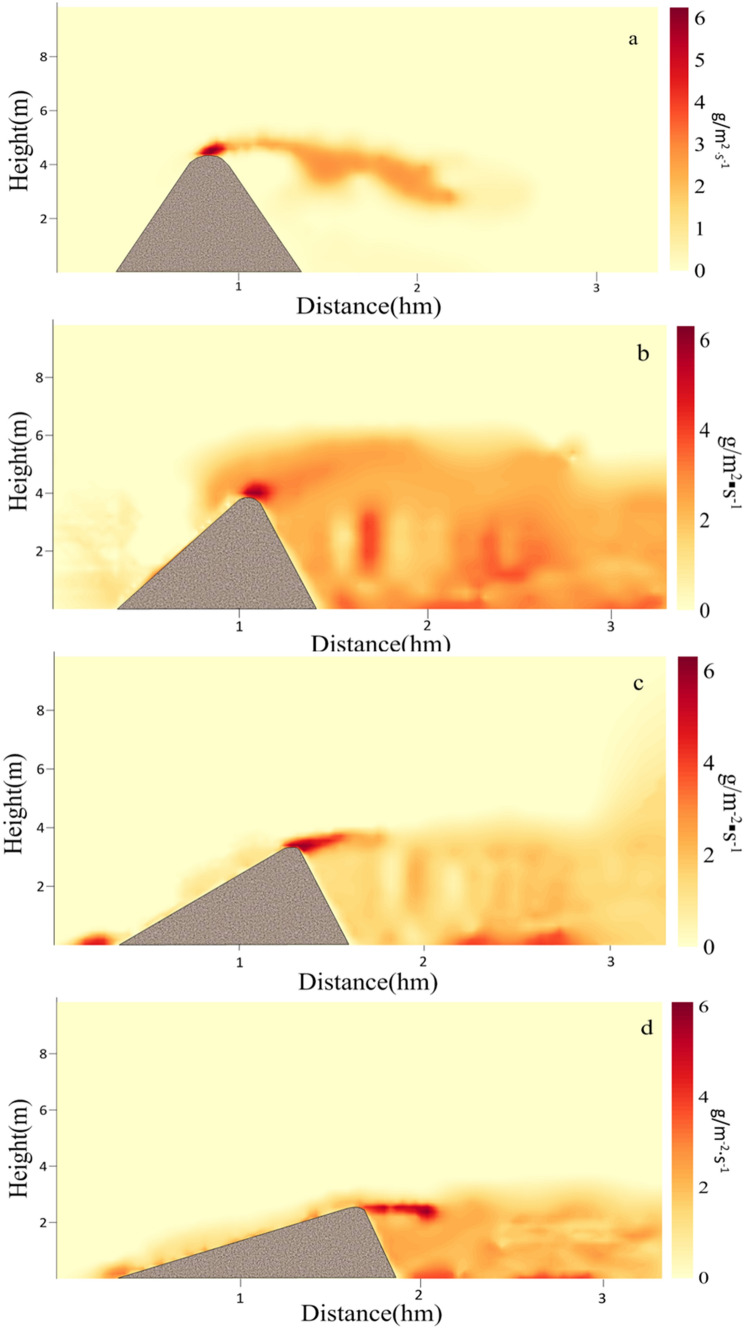
Spatial variation of two-dimensional sand flux around the dune at four stages in Fig. 1.

Figure 4 simulated the changes of sand flux around the central axis of the four stages during the evolution into a crescentic dune. Figure 4a showed the distribution of sand flux after loading wind flow at the initial stage. At this stage, the sand flux at the windward crest was the largest. Then the sand particles after being blown to the leeward side diffused in the downwind direction, and the depositing dust would be affected by the vortexes at the leeward side, and then the finer particles would migrate in the direction of vortex rotation.

Figure 4b revealed the sand flux of the dune at the time of evolution to the second stage. We found that although the sand flux at the windward crest was always the largest, the sand flux around the whole area in the downwind behind the leeward side had become larger from at this stage. During this period, the range of sand flux was the largest and the amount of sand flux was also the largest, and the amount of sand flux in many positions had reached 4 g·m^−2^ s^−1^. At the same time, the amount of sand lifting near the ground was also very large. In addition, the windward crest was still the most significant position of instantaneous sand flux.

Figure 4c displayed the change of sand flux during the evolution to the third stage. It showed that the maximum sand flux was still maintained at the windward crest, but the direction of sand transport there was more horizontal. As the height of the dune decreased, the wind velocity at the windward crest also decreased. However, due to the increase of sand density in the region of leeward side, the collision of the sand particles would lead to the increase of the shear stress of the impacted sand particles in the air and on the ground. Although the velocity of the original sand particles decreased after the collision, it would continue to gain acceleration energy through the wind flow. Especially when the sand particles in the turbulence of wind flow, it was easier to obtain continuous energy supply^55^. The whole process would increase the migration distance of sand particles and then increased the sand flux^56,57^ on the ground. The decrease of the height and slope of the windward side made the difference between the windward side and the wind direction smaller, so the acceleration effect of the sand particles decreased, but the amount of sand flux in the horizontal direction would increase.

As shown in Fig. 4d, when the dune evolved to a more typical crescentic shape, the sand transport at the windward crest almost showed horizontal movement, while the phenomenon of the sand lifting on the surface of the whole windward side was obvious, and the amount of sand flux was also larger. As the slope decreased, the movement of sand particles on the windward side changed from the windward crest of the initial stage to the windward toe now, so the sand transport tended to occur on the whole slope. At this stage, the volume of vortexes behind the leeward side was compressed and reduced, and the shear stress of the eddy current to the sand of the leeward side and that of the leeward toe was more uniform. Therefore, the distribution of sand lifting or sand settlement at the position of the downwind behind the leeward side was more uniform.

**Figure 5:**
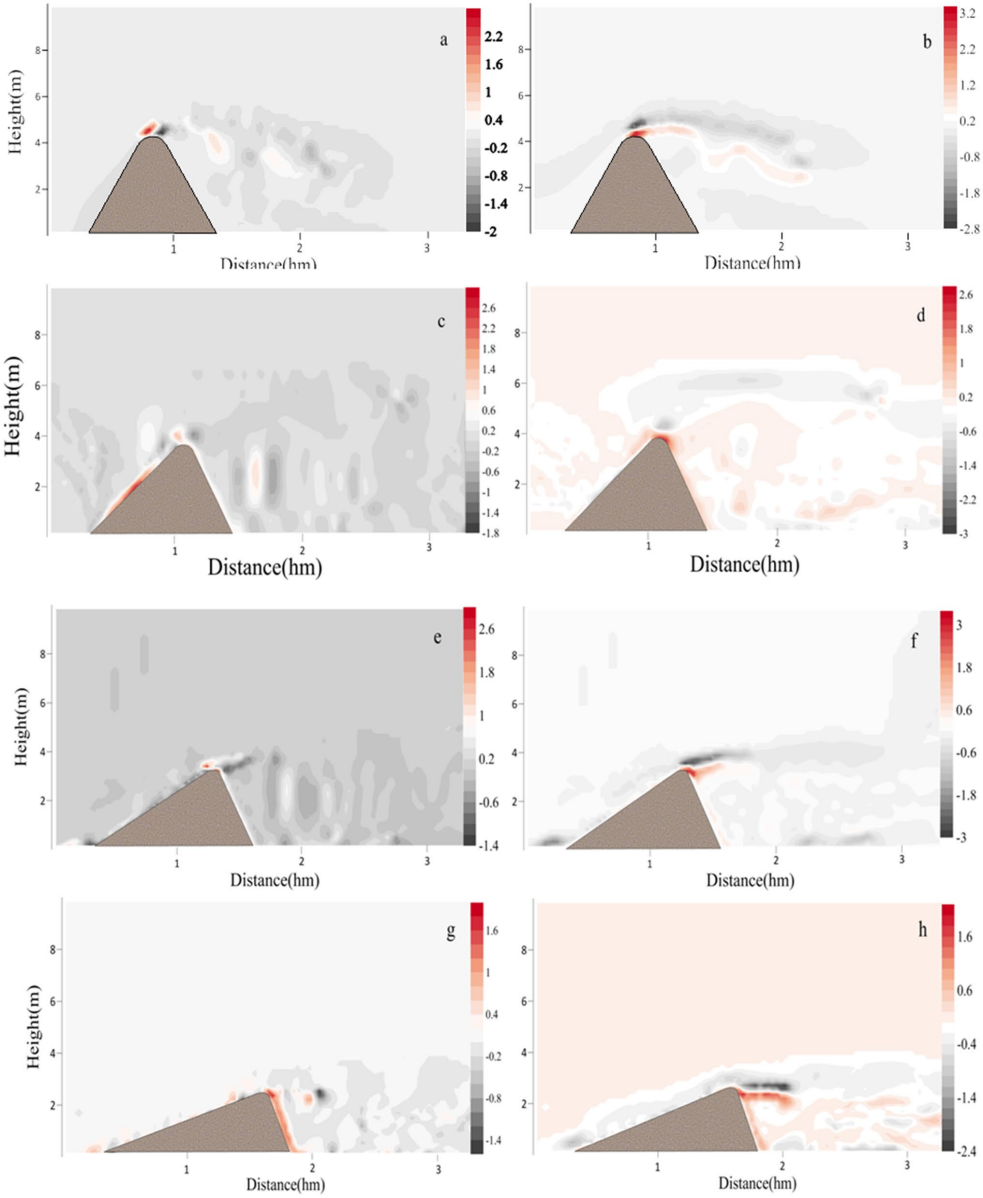
The horizontal (**a**,**c**,**e**,**g**) and vertical (**b**,**d**,**f**,**h**) components of spatial sand flux at different stages in Fig. 4.

Figure 5 showed the horizontal and vertical components of spatial sand flux in different stages. The positive and negative values in the horizontal direction indicated that the direction of sand migration was the same or opposite of the wind direction, respectively. The positive and negative values of vertical direction indicated that the direction of sand movement was rising or falling, respectively. From Fig. 5a,b, we found that it had the maximum horizontal and vertical sand flux on the windward crest at the beginning stage of simulation. Then at the second stage of simulation, Fig. 5c displayed that the horizontal sand flux on the windward side was relatively large, while that on the leeward side was not large. Figure 5d revealed that most of the sand particles jumped in the surrounding space below the peak of the whole dune, indicating that the transport of wind flow to sand flow on the ground was stronger at this stage. From the horizontal transport in Fig. 5e, it could be seen that the sand flux on the windward side was relatively stable and the amount of sand flux was small, but there were still more negative sand flux on the leeward side. Figure 5f discovered that the sand particles showed more settlement at this stage, indicating that the sand lifting effect of leeward side was reduced. The exhibition of Fig. 5g,h reflected that with the decrease of the height of dunes, the sand flux of the whole dunes decreases. Although Fig. 5h showed that the eddy current on the leeward side increased the sand flux in vertical direction, the sand flux was smaller in horizontal and vertical direction compared with the stages in Fig. 5c,d. Because tall dunes are more likely to cause small eddy currents near large ones, thus increasing sand flux on the ground and on the surface of dunes. For small dunes, the opposite is true.

**Figure 6:**
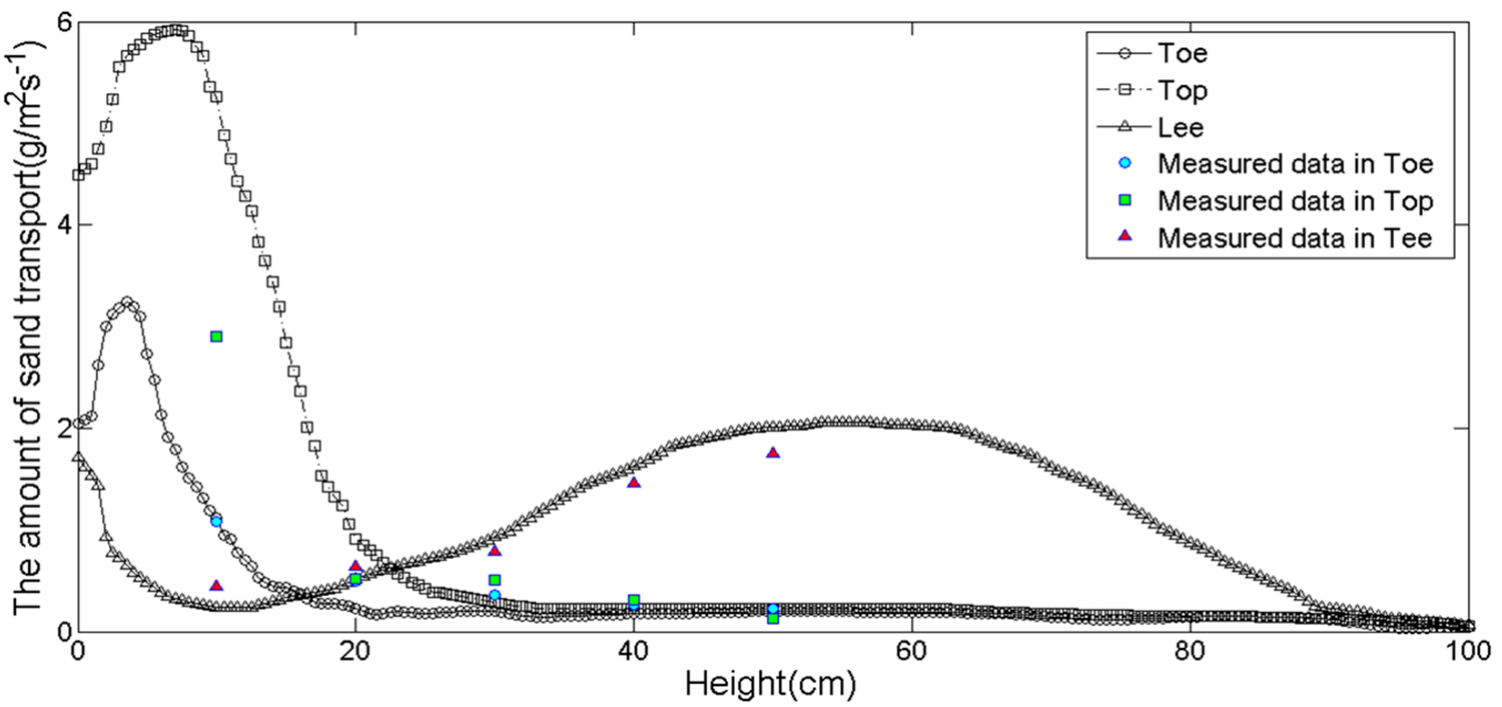
Comparison between the simulated and measured values of sand flux in final form.

To verify the accuracy of simulated sand flux, we compared the measured sand flux in three different locations of measured dune in Minqin County (Fig. 6). The relevant data of the measured dune and the velocity of Fig. 2 came from the same dune. We found that the simulated values and measured values of sand flux were in good agreement with the trend at the toe of the sand dune and the leeward side, but the difference was great at the windward crest. The reasons for the difference were as follows: firstly, there was a certain difference in the scale between the actual sand dune we used to compare and that we used to simulate. Secondly, we used a stable wind field in the simulation, but in practice it was a pulsating wind field. In the introduction of the method, we had emphasized that the input of pulsating wind field in the simulation would lead to the obvious fluctuation in the simulated sand flux, which was also the difficulty of correcting the model.

### Two-dimensional and three-dimensional morphological changes of sand dunes at different stages

**Figure 7:**
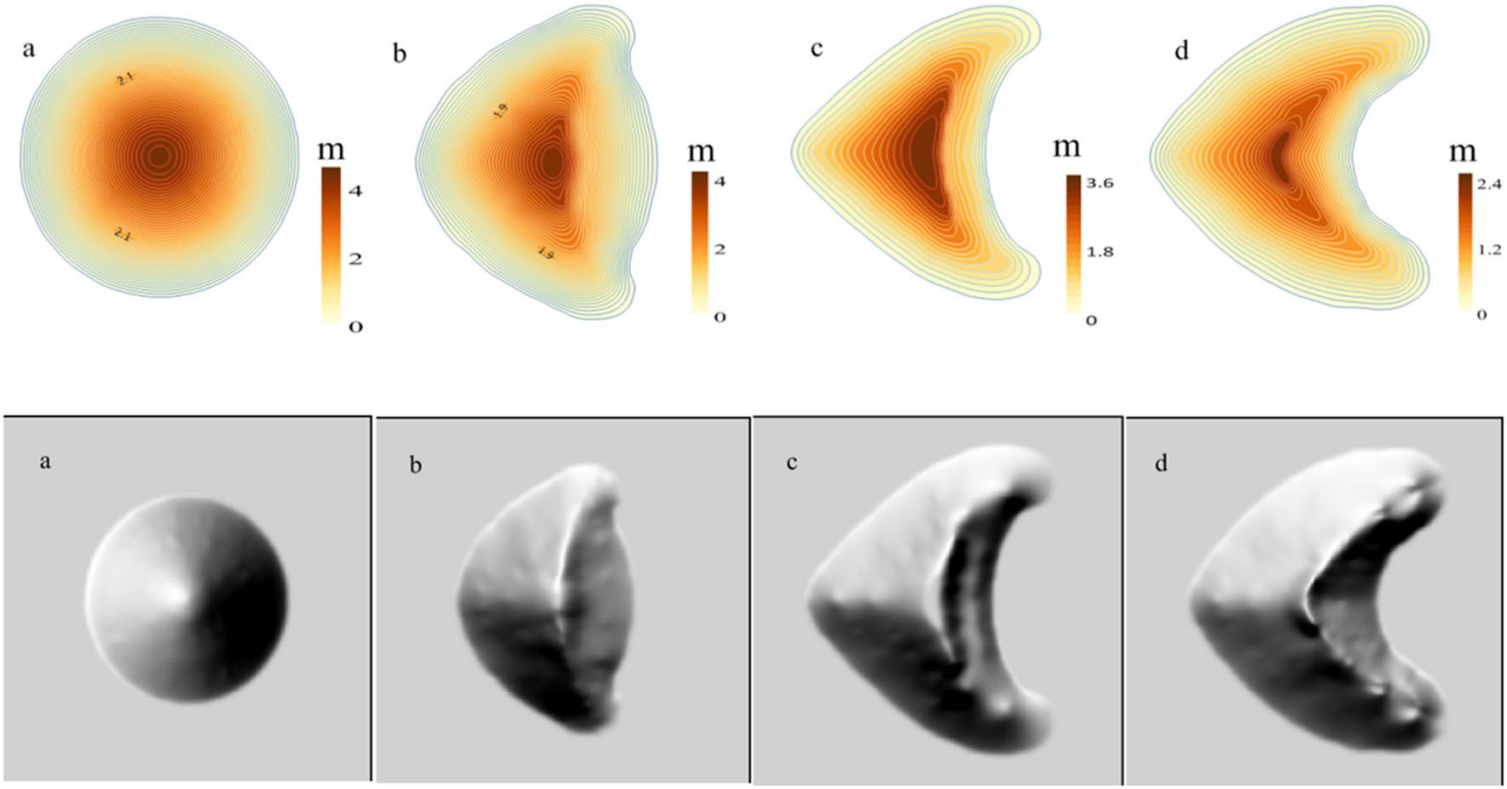
Morphological evolutions of sand dunes at different stages. The map was plotted using Surfer 16 (https://www.goldensoftware.com/).

Based on the simulated wind field and sand flux, we constructed the morphological evolution of crescentic dune. The evolution time of dune from the initial morphology to the last three stages was 11.5, 20.7 and 32.2 days, respectively. Obviously, the evolution time was less than the actual formation time (several weeks or one month) of a crescentic dune of similar scale. The reason was that the model set the continuous wind velocity and the constant stable state of parameters, which accelerated the evolution of dune. After 11.5 days of unidirectional wind action from the initial form of Fig. 7a, the circular dune gradually formed two short horns, thus initially having the morphology of the crescentic dune (Fig. 7b). At this stage, the windward side and the brink parabola were obvious, and the range of the windward crest was more concentrated, but the highest point and the brink parabola did not coincide. The windward side was relatively smooth, and there was still a large protruding behind the leeward side. Then after 20.7 days of evolution, the windward and leeward sides became more pronounced, and the range of the windward crest was elongated with the lengthening of both horns. Then the windward side began to become steep and formed windward toe, and the back of the leeward side had been tightened in a curved moon shape (Fig. 7c). After another 32.2 days, the dune reached the final evolutionary form, the windward crest coincided with the brink parabola, and the leeward side became more curved as the horns stretched (Fig. 7d).

Because of the long simulation time, we tried to obtain the rapid morphological evolution process of dune by increasing the amount of sand flux or reducing the diameter of sand particles. However, the final morphology of the dunes obtained by these two methods was not the same. Because the increase in amount of sand flux needed to increase the wind velocity, it would inevitably lead to the long distance extension of the horns of the crescentic dune. And the movement distance of the sediment and the amount of sediment in the unit range on the leeward side would change. While the method of reducing the diameter of sand particles would also make the deposition and drift of sand particles obviously different, especially the characteristics of deposition in the downwind behind the leeward side would become more complex, and the sand particles could even be deposited far away. These changes further indicated that the migration and change of sand dune were affected by many factors. In the simulation, we could fit the reasonable results by adjusting the individual parameter, but the changes in the actual situation were the results of the complex action of many factors together.

**Figure 8:**
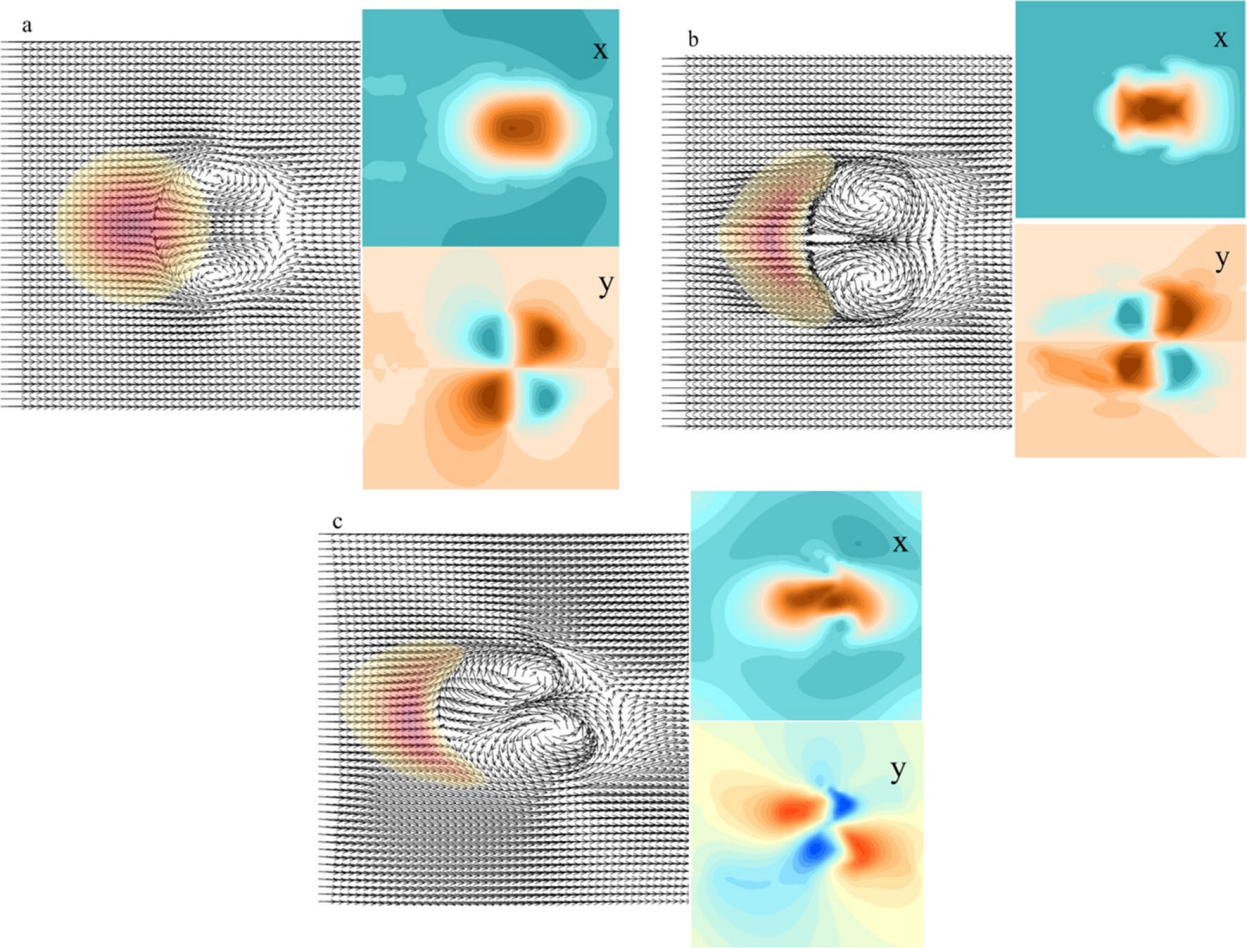
The simulated wind flow field distribution around the circular dune (**a**); the simulated wind flow field of the symmetric crescentic dune and the velocity component of the horizontal and vertical (x and y) directions (**b**); the simulated wind flow field around the asymmetric crescentic dune and the velocity component of the horizontal and vertical (x and y) directions (**c**). The map was plotted using Matlab 2014a (https://www.mathworks.com) and Surfer 16 (https://www.goldensoftware.com/).

The simulation results of Fig. 8a showed that two vortexes appeared on both sides of the leeward side of the initially circular dune, which pushed the sand particles in the inner side of the dune to the opposite direction of the wind, and pushed the sand flow at the edge of the dune to the downwind direction, and the wind on both sides continuously gave the rotation power to the vortexes as its outer boundary, which lead to the extension of the two horns of the crescentic dune. Figure 8b revealed that after the formation of crescentic dunes, there were still two vortexes in the leeward side. From the direction of the vortexes, we found that the sand particles of the leeward side were distributed to both sides of the dunes and gradually the horns developed with the transmission of the sand particles. Under the influence of this wind flow, although the dunes moved slowly backward as a whole, the sand particles on the leeward side moved slower than that of the two horns, so it gradually formed the crescentic shape of dune with the two horns downwind direction extending and the leeward side gradually depressed. It was the existence of these two vortexes that made the horns extending continuously, but when the length of the horns reached a certain length, that is, when the vortexes formed in the leeward side could not act on the sand flow on the horn, the horn might produce new small crescentic dunes under the continuous action of the wind (Fig. 8c).

### Effect of wind velocity and air viscosity on sand flux

To test the influence of different parameters on sand migration and sand flux, we simulated the morphological changes of dune obtained under different wind velocity and Reynolds numbers, respectively. Where the wind velocity increased from the original 10 m s^−1^ to 12 m s^−1^, the air viscosity changed from the original 1.46 × 10^–5^ m^2^ s^−1^ to 2.46 m^2^ s^−1^.

**Figure 9:**
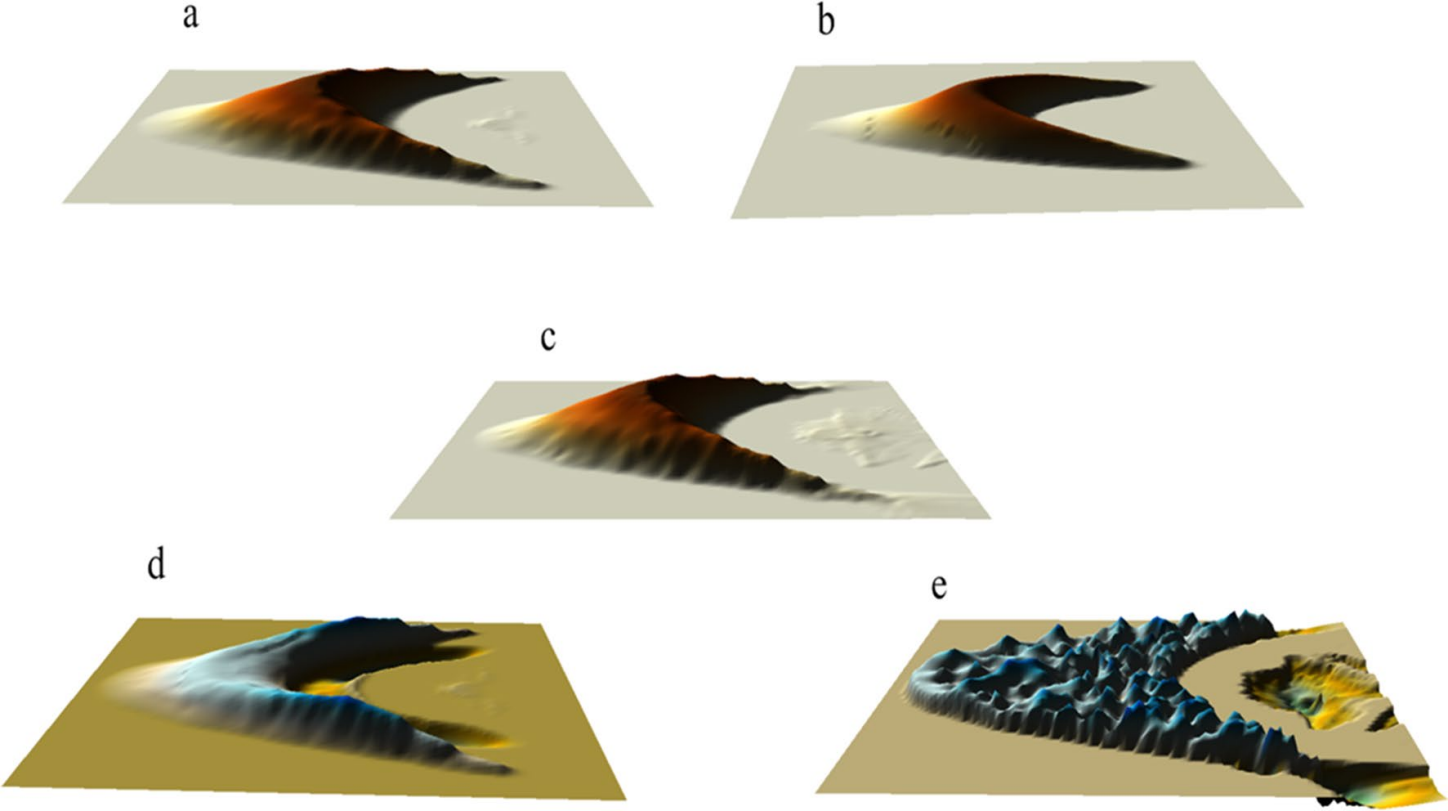
Simulation of the sand distribution on the leeward side in several cases. (**a**) At the wind velocity of 10 m s^−1^; (**b**) high Reynolds; (**c**) at the wind velocity of 12 m s^−1^; (**d**) difference between (**a**) and (**b**); (**e**) difference between (**a**) and (**c**). The map was plotted using Surfer 16 (https://www.goldensoftware.com/).

Figure 9a was the result of sand dune generated after 1.26 × 10^8^ iterations with simulated wind velocity of 10 m s^−1^. The actual time of simulation was approximately 35 days. Figure 9b was the result of sand dune obtained after the iteration of the same time when the maximum wind velocity was set to 12 m s^−1^. The comparison between the two pictures showed that the larger wind velocity made the sand migration faster. Figure 9d revealed the difference between Fig. 9a and Fig. 9b. From which it could be seen that the increase of wind velocity increased the migration velocity of sand dune. And the extension direction of the two horns of the sand dune also showed a trend of more convergence behind the windward side.

Figure 9c displayed the change of sand deposition after changing Reynolds number. From this figure we found that the sand deposition behind the dune increased obviously. Figure 9e showed the difference between Fig. 9a and Fig. 9c. From which we could see that the change of sand particles on the surface of sand dune was extremely uneven, and the situation of sand lifting and deposition was more complicated, especially the range in change of deposition behind the leeward side was very large. These results indicated that the increase of air viscosity had a significant effect on the deposition distribution of sand particles on the leeward side, especially the deposition amount in the leeward toe and the middle part. In the previous section, we discussed the low-pressure vortexes caused by the wind flow field in the downwind behind the leeward side (Fig. 3). The shear stress produced by the friction between vortexes and the ground may increase the sand flux^11,41,58^, because the increase of the number and the intensity of vortexes in the air flow all can increase the shear stress of the ground surface^59,60^. Moreover, the change of air viscosity have a strong effect on the spatial turbulent intensity^51,61^. Then the change of air viscosity further influenced the distribution of sediment by affecting the spatial turbulent intensity. Therefore, it is for this reason that the large difference of diurnal temperature in desert area will affect the change in sand flux of sand dunes^62^.

### Effect of height of sand dune on shear stress and turbulent intensity behind the leeward side

We simulated the wind flow field and sand flux in the evolution of the dune, the results showed that with the changes of the height of the dune, the sand flux of the whole dune changes significantly, especially the turbulent intensity and the number of vortexes at the leeward side also affected the variation of sand flux. In the simulation, when the wind field and sand flux were calculated to reach a stable state, we regarded it as the steady-state result in the current situation. The end condition of the iterative physical quantity was set as follows:

26 $$\frac{{2\|u^{\prime} - u^{^{\prime} - 1}\| }}{{ \|u^{\prime} - u^{^{\prime} - 1} \|}} < 10^{ - 2}$$

**Figure 10:**
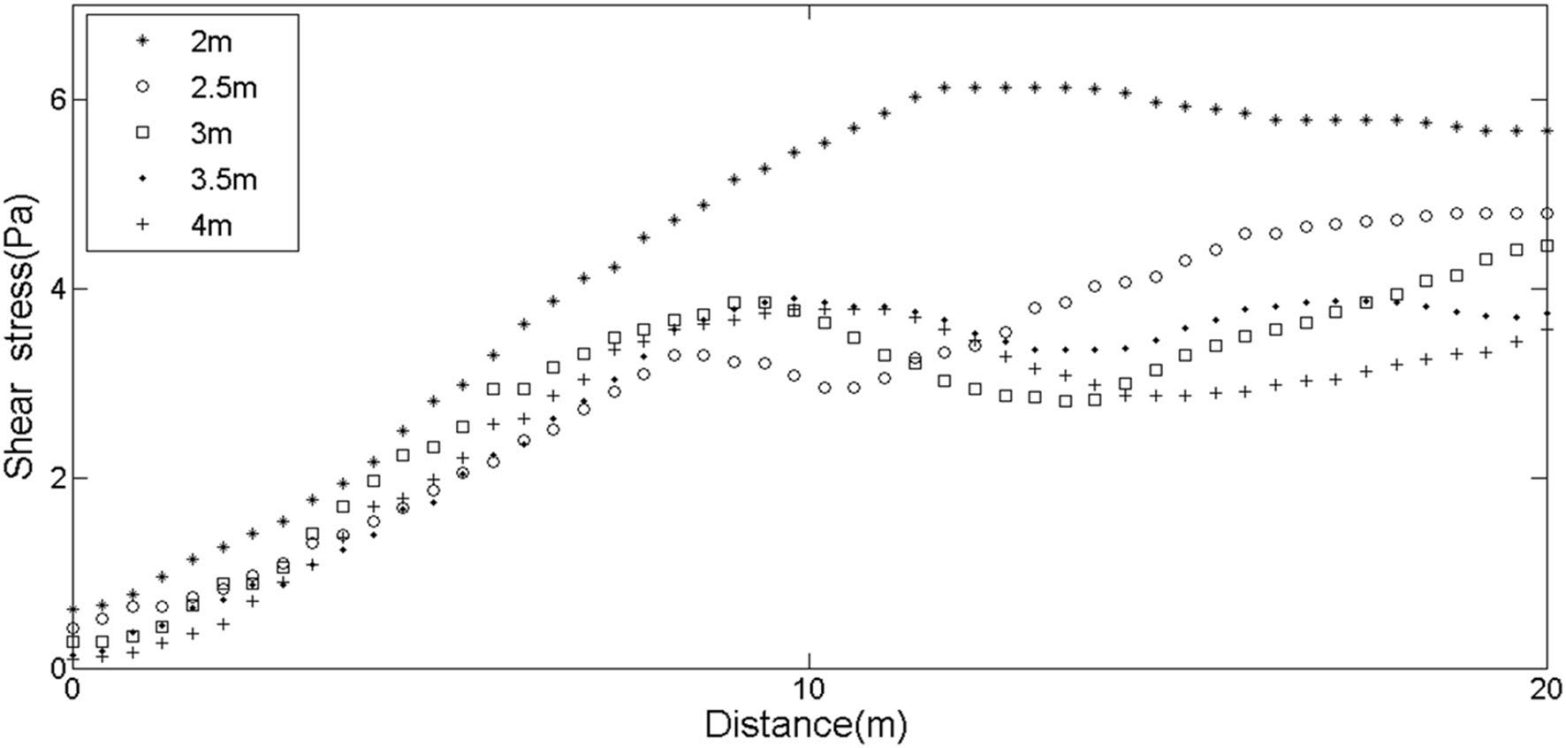
Changes of shear stress on the ground surface corresponding to different distances behind the leeward side at different heights of sand dune.

Through simulation, we found that except that the sand dune with a height of 2 m had a great influence on the shear stress of the ground surface behind the leeward side, the increases of other height of sand dune had no obvious effect (Fig. 10). But when the shear stress was stable below 6 Pa, the disturbance of shear stress decreased with the increase of height. Therefore, for dunes with a height of less than 4 m, the effect of wind flow on shear stress was not significant. This was not consistent with the results of the simulated sand flux in Fig. 3. Figure 3 suggested that the deposition of sand particles behind the leeward side of sand dune with higher height was more uneven. The reasons were as follows: firstly, the position to lift sand on the leeward side was higher and the deposition range was large. And secondly, the vortexes behind the leeward side of tall dune were not a single distribution. If there was an irregular air flow gap between the middle part and the two horns of dune, it was possible to form more low pressure zones, thus forming more vortexes, which would increase the amount of sand flux on the whole dunes. Then the changes would result in changes of shear stress behind the leeward side, triggering a wider range of sand particles on the ground. However, the vortexes in the downwind behind the leeward side of low dunes was smaller and more stable, and the stable vortexes would form more stable deposition in the leeward side. Therefore, the morphological changes of low sand dunes were also relatively stable during the migration process. These results suggested that the wind velocity did not completely determine the magnitude of shear stress on the ground surface, and other factors such as direction of eddy current and fluctuation of air flow all could affect the sand flux.

In order to analyze the influence of height changes of sand dune on the turbulent intensity behind the leeward side, we used the following formula to calculate the turbulent intensity^63,64^:

27 $${\text{E}}_{{{\text{intensity}}}} = \sqrt {{\text{u}}^{^{\prime}2} } /{\text{U}}_{{{\text{h}} = 0.25}}$$

where $u^{\prime}$ was the instantaneous velocity of the wind flow.

**Figure 11:**
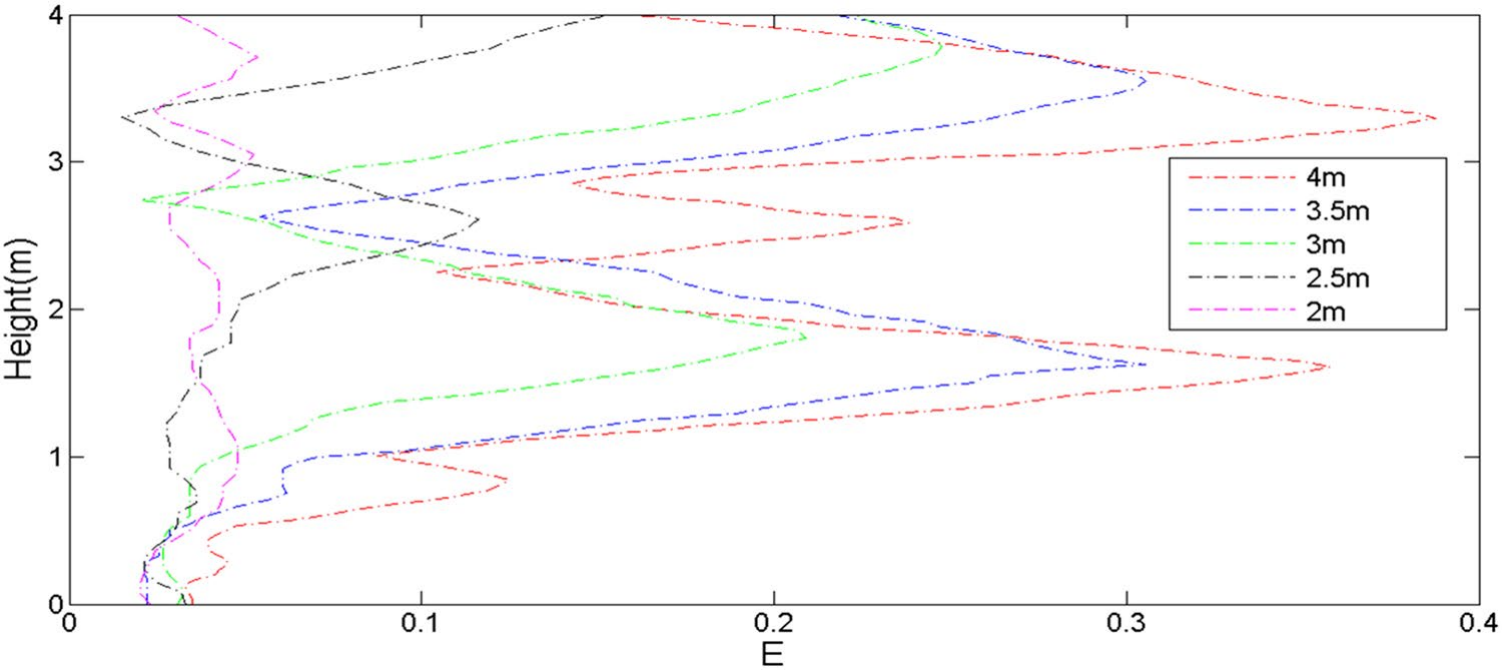
Turbulent intensity at the leeward toe of the sand dunes with different heights.

Figure 11 indicated that the sand dune with height of 4 m had larger turbulent intensity at two positions, 1 m and 3 m. Moreover, the closer almost every dune was to the ground, the smaller the turbulent intensity. One of the reasons was related to the wind velocity, and the other was related to the fluctuation of the air flow, that is, the more complex the fluctuations and turbulence, the larger energy the fluid generated. According to the results of Figs. 10 and 11, it could be concluded that the magnitude of shear stress was not proportional to the turbulent intensity, which indicated that the calculated shear stress and turbulent intensity could only represent the intensity of sand transport to a certain extent. After all, there was a complex relationship between the amount of sand flux and wind velocity, size of sand particles and the flow direction between vortexes.

## Conclusion

On the basis of the RA-NS equation and the stress and sand flux model, the wind field diagram of a circular dune with a height of 4.2 m and a length of about 100 m during the four evolution periods of the evolution into a crescentic dune was simulated in this study. Based on the relationship between wind shear stress and sand flux, the two-dimensional distribution of sand flux in the main axis of sand dune was obtained. By comparing with the measured velocity, we verified that the simulated velocity of wind field and the simulated sand flux at different positions and heights behind the leeward side were all accorded with the measured results. From the simulation results, we found that the variation of dune height may lead to different vortex patterns on leeward slope, the complexity of eddy current behind the leeward side had an important influence on the sand flow of the leeward side and the transmission and migration of the sand particles on the ground. And the increase of wind velocity increased the sand flux of the main body of the sand dune. In addition, the influence of the height of the dune on the turbulent intensity of the leeward side was very significant, and the turbulent intensity increased with the height of the dune. Moreover, the changes of height of tall sand dunes had significant effect on the surface shear stress caused by eddy current behind the leeward side, but the changes of height of low sand dunes had little effect. This was because the leeward side of tall dunes was more prone to steep slope, and there was also more space to generate more vortexes and more friction with the surface. The leeward side of low dunes was relatively slow, and the gap between the vortex boundary and the leeward side was less than enough to produce more vortexes. These phenomena were due to changes in special sand flux during the evolution of dunes.

By comparing the relationship between surface shear stress and turbulent intensity of sand dunes with different heights, it could be seen that on the basis of increasing surface shear stress by wind velocity and turbulent intensity, the fluctuation of wind velocity and eddy current caused by vortexes were also the reasons for increasing sand migration distance.
